# Use of Natural Language Processing to identify Obsessive Compulsive Symptoms in patients with schizophrenia, schizoaffective disorder or bipolar disorder

**DOI:** 10.1038/s41598-019-49165-2

**Published:** 2019-10-02

**Authors:** David Chandran, Deborah Ahn Robbins, Chin-Kuo Chang, Hitesh Shetty, Jyoti Sanyal, Johnny Downs, Marcella Fok, Michael Ball, Richard Jackson, Robert Stewart, Hannah Cohen, Jentien M. Vermeulen, Frederike Schirmbeck, Lieuwe de Haan, Richard Hayes

**Affiliations:** 1 Kings College London, Institute of Psychiatry, Psychology, and Neuroscience, London, United Kingdom; 2 University of Taipei, Department of Health and Welfare, Taipei City, Taiwan; 3 South London and Maudsley NHS Foundation Trust, London, United Kingdom; 4 University of Amsterdam, Department of Psychiatry, Amsterdam, The Netherlands; 5 Arkin Institute for Mental Health, Amsterdam, The Netherlands

**Keywords:** Literature mining, Epidemiology

## Abstract

Obsessive and Compulsive Symptoms (OCS) or Obsessive Compulsive Disorder (OCD) in the context of schizophrenia or related disorders are of clinical importance as these are associated with a range of adverse outcomes. Natural Language Processing (NLP) applied to Electronic Health Records (EHRs) presents an opportunity to create large datasets to facilitate research in this area. This is a challenging endeavour however, because of the wide range of ways in which these symptoms are recorded, and the overlap of terms used to describe OCS with those used to describe other conditions. We developed an NLP algorithm to extract OCS information from a large mental healthcare EHR data resource at the South London and Maudsley NHS Foundation Trust using its Clinical Record Interactive Search (CRIS) facility. We extracted documents from individuals who had received a diagnosis of schizophrenia, schizoaffective disorder, or bipolar disorder. These text documents, annotated by human coders, were used for developing and refining the NLP algorithm (600 documents) with an additional set reserved for final validation (300 documents). The developed NLP algorithm utilized a rules-based approach to identify each of symptoms associated with OCS, and then combined them to determine the overall number of instances of OCS. After its implementation, the algorithm was shown to identify OCS with a precision and recall (with 95% confidence intervals) of 0.77 (0.65–0.86) and 0.67 (0.55–0.77) respectively. The development of this application demonstrated the potential to extract complex symptomatic data from mental healthcare EHRs using NLP to facilitate further analyses of these clinical symptoms and their relevance for prognosis and intervention response.

## Introduction

The increasing use of electronic health records (EHRs) across health services provides opportunities for research using real world data^1^. However, there are challenges to realizing the potential of EHRs in research. For example, whilst some information is recorded in structured fields, often useful contextual information, such as description of symptoms, is embedded in free text^2^. Information from free text fields can be extracted and coded manually to generate datasets which can be analyzed for research, but this is not feasible on a large scale. Consequently, the advantages of conducting studies on a larger scale are lost: for example, the statistical power to look at rare exposures or outcomes, and the ability to control for multiple potential confounders. An alternative to manually coding free text fields is to develop automated approaches through the application of Natural Language Processing (NLP).

NLP approaches have been used previously to facilitate a number of studies using EHRs^3^, although the technique is still at a relatively early stage of application. NLP algorithms take into account the linguistic context around words and phrases of interest and go beyond a simple keyword search of the text: for example, distinguishing between instances where a patient is described as experiencing a particular symptom from instances where the texts states that the patient is not experiencing that symptom, or where it is someone else (e.g. a friend or relative) who is experiencing that specific symptom. A key word count would not be capable of making such distinctions. NLP applications can be developed using machine learning and rules based approaches, each with its own advantages and drawbacks in relation to specific problems.

Machine learning in the context of NLP refers to an automated method of creating an NLP application^4^. It involves an annotated set of training data being utilized by various algorithms to create models to classify future documents^5^. The time taken for the algorithm to create this model and to apply the model to future issues varies based on a wide range of factors; however, a machine learning approach takes substantially less time to develop than a rules-based approach.

In contrast, rule based approaches involve a human coder manually analyzing training data and creating rules based on their observations of the data^6^, these rules are then implemented programmatically. This can be a challenging task, as the rules created need to be broad enough to ensure that they are applied to all the required instances, but not excessively broad as might lead to the rules being incorrectly applied. An advantage to the rules based approach is that it, arguably, allows for rules to be created for substantially more complex problems than most machine learning algorithms would be able to solve (particularly with similarly sized datasets)^7^.

NLP applications can operate with a high degree of precision (positive predictive value) and recall (sensitivity) although this can vary considerably between applications^8^. Variation in performance of NLP applications may relate in part to the complexity of the task being undertaken^9^. As EHRs continue to be exploited for research, NLP is being applied to increasingly subtle and complex tasks^10^.

In this paper, we describe the development of an NLP application for extracting data on obsessive compulsive symptoms (OCS) and obsessive compulsive disorder (OCD) from free text in EHRs in patients with schizophrenia, schizoaffective or bipolar disorders, using a rules-based approach. In the context of schizophrenia, OCS can be defined as persistent, repetitive, intrusive, and distressful thoughts (obsessions) not related to the patient’s delusions or repetitive, goal-directed rituals (compulsions) clinically distinguishable from schizophrenic mannerisms or posturing^11^. For the purposes of this investigation we define OCD patients as a subset of OCS patients where a clinician has recognised that these symptoms are of sufficient severity, duration, or cause sufficient functional impairment that a clinical diagnosis of a disorder is given. OCS are relatively common in schizophrenia or related disorders and are associated with depressive symptoms and poorer functioning^12^. There are a number of challenges inherent in identifying co-morbid OCS from clinical record for this patient group. For example, the terms used to describe OCS (such as obsession and compulsion) are not specific to these symptoms and these symptoms need to be distinguished from other aspects of these disorders. This study provides an example of the development of an NLP algorithm for a relatively complex task using a large psychiatric EHRs database. We describe obstacles and solutions.

## Methods

### Setting

The data used to develop the NLP algorithm for extracting OCS were obtained from the South London and Maudsley NHS Foundation Trust (SLaM) which is a near-monopoly secondary mental healthcare service provider to 1.36 million residents in four boroughs of south London (Croydon, Lambeth, Lewisham and Southwark), as well as providing some national tertiary mental healthcare services. The SLaM Biomedical Research Centre (BRC), supported by National Institute for Health Research funding, provides anonymised electronic clinical records from the SLaM Case Register for research purposes through the BRC Clinical Record Interactive Search (CRIS) system. The CRIS system was developed in 2008 and accesses full EHRs across all SLaM services since 2007, including both structured and open-text fields, currently on more than 300,000 service users. A detailed description of CRIS and its development is described elsewhere^7^.

### Ethics statement

The CRIS data resource received appropriate research ethics approval as a de-identified database for secondary analyses from Oxford Research Ethics Committee C (reference 08/H0606/71 + 5) and the authors can confirm that the study presented here was performed in accordance with the guidelines and regulations set out in this approval.

### Inclusion criteria

To develop and test the OCS NLP algorithm, data extracts were obtained for individuals who were aged 15 years or older at the time of their first severe mental illness (SMI) diagnosis date within the observation period (from 1 January 2007 to 31 December 2015) and who had received a diagnosis (ICD-10 code) of schizophrenia (F20), schizoaffective disorder (F25), or bipolar disorder (F31) during the observation period. Diagnoses were obtained from structured fields and also from unstructured free text using a previously validated NLP algorithm, described elsewhere^13^.

### Definition of OCS

As mentioned above, in this study OCS were defined according to the Structured Clinical Interview for DSM Disorders–Patient (SCID-P)^14^ as ‘persistent, repetitive, intrusive, and distressful thoughts (obsessions) not related to the patient’s delusions, or repetitive, goal-directed rituals (compulsions) clinically distinguishable from schizophrenic mannerisms or posturing”. As such, individuals whose obsessional thoughts or compulsions were related to psychotic content of thoughts or delusions were not considered to have comorbid OCS^9^.

### Extracting data for training and validation of the algorithm

Data were extracted from EHRs for training and development of the algorithm from those individuals who met the inclusion criteria. To avoid reading and coding a substantial volume of unrelated documents we applied a filter such that we only extracted documents containing specific key terms. Although, once developed, an NLP algorithm can be substantially more sophisticated than a key word search, the development process may include keyword searchers. In this instance a set of key terms were selected which were potentially broad enough to cover all the records that mentioned OCS. The Yale Brown Obsessive Compulsive Scale (Y-BOCS)^15^ was used as a guide to select these key words. The following key words terms (as shown in table 1) were used to filter the EHRs:

- OCD (and variations such as O.C.D)
- Obsess (and variations such as “obsessional” and “obsessive”
- Compulsive (and variations such as “compulsion”, “compulsiveness” and “compelled”)
- Ritual (and variations such as “ritualistic”)
- Hoard (and variations such as “hoarding” or “hoarded”)
- The presence of any of the YBOCS key terms.
- The presence of any of the Patient Insight Key terms.

Through applying the filter, a random sample of 900 documents that contained at least one of the terms shown in Table 1 (including patient notes and correspondence), with one document per unique patient, were randomly extracted from the anonymised EHRs. This sample was then divided into a training set (600 documents) and a validation set (300 documents). These documents contained multiple instances of references to OCS, with each document containing at least one instance, with no upper limit. Text strings around each key word (described above) were extracted from these documents. Each text string included the keyword and the sentence which contained this key word plus two sentences either side of the key word sentence. This was to ensure that any contextual information contained in the surrounding sentences could be incorporated into the NLP algorithm. In some instances, the text strings comprised fewer than five sentences due to there being less than two sentences before and/or after the keyword sentence in that particular document.

**Table 1:** Key modifier words used in the natural language processing application for OCS.

OCS Keywords	YBOCS Keywords	Patient Insight Keywords
Obses* (Includes variations such as ‘obsessive’ and ‘obsessional’)	Clean* (Includes variations such as ‘cleaned’ or ‘cleanliness’)	Distres* (Includes variations such as distressed or distressing)
Compul* Includes varations such as Compulsive or compulsively, but specifically excluding “compulsory”.	Wash* (Includes variations such as washing or washed)	Unwanted
OCD* (Includes variations such as OCD and O.C.D)	Check* (Includes variation such as checking and checked)	Repugnant
Hoard* (Includes varations such as Hoarding and Hoarded)	Repeat* (Includes varations such as repeatedly or repetitive)	Repulsive
Ritual* (Includes variations such as ‘ritualistic’ and ‘ritually’)	Count* (Includes variations such as counted or counting)	Egodystonic
	Order* (Includes variations such as ordered or ordering)	Intrusive
	Counting	Intruding
	Rearrange*(Includes variations such as rearranging or rearranged)	Unable to stop

### Developing manual coding rules

The training and validation sets of documents were then manually coded according to a predetermined set of manual coding rules which were developed using the Y-BOCS as a guide. An approach taken when developing the manual coding rules for identifying OCS in text strings has been outlined in appendices A and B. For example, if the text mentioned that the patient had both obsessions and compulsions, then the patient was classified as having OCS. However, if the text only mentioned obsessions or compulsions (but not both terms) this was only considered OCS if the text also listed specific examples found in the Y-BOCS, such as checking or cleaning, or described intrusive, ego-dystonic thoughts. There were a number of reasons for this conservative approach: firstly, clinical text may be produced by a range of different health professionals or may describe a patient’s belief about themselves and secondly, the terms obsession or compulsion are used in a wide range of contexts beyond OCS.

### Annotating training and validation data sets

The training dataset consisted of 600 documents (containing at least one of the key words) which were manually annotated by two annotators (DA, RH) individually annotating each of the records. After the annotations were completed, the results were compared, and individual points of disagreement were identified. To resolve these points of disagreement, a discussion occurred between the two annotators, under the supervision on an arbitrator (DC) to ensure that the process did not give one of the annotators an undue level of input. Inter-annotator reliability between the two annotators produced observed agreement of 92.0% (Cohen’s *κ* of 0.80), indicating good inter-annotator agreement in determining the OCS coding rules.

### Development of an NLP algorithm for extracting OCS

The training data were used to create classification rules needed to build the algorithm. The algorithm was developed using Generalized Architecture for Text Engineering (GATE). which includes a suite of tools for the development of NLP rules which are based on JAPE. JAPE is a unique, Java based, NLP scripting language that is native to GATE. It allows users to generate rules with very high levels of complexity. The manual coding rules which had been applied to annotate the training set were combined with observations of the annotated training set data to create a set of broad rules in JAPE which were then integrated into the application. This involved developing sets of exclusion rules and inclusion rules. Inclusion rules, determined the patterns of text required for an instance to be classed as positive, in the absence of exclusion rules. Exclusion rules used sets of exclusion terms, which would lead to an instance being classed as a negative (these are terms such as negations, or experiencers that are not the patient themselves). The algorithm involved the following steps (Table 2 contains terms that were used in the exclusion of terms as described in steps 4–8)

1. Splitting the text on a sentence by sentence level
2. Finding the presence of a possible OCS reference (in the context of the particular app) within the text.
3. Check for a combination of terms that would indicate an instance of OCS, as in the context of the particular app (e.g. the Hoarding app identifies an instance of Hoarding as an OCS symptom).
4. Exclude all instances wherein the text was characteristic of prompt questions within a clinical questionnaire. Specifically, the algorithm identified all combinations of words and punctuation that were unique to forms (which were determined through analysis of the training data). If any instances contained any of these combinations, they were excluded. In the context of the extracted data, there were very few cases of these.
5. Exclude any instances wherein the sentence contains any negating terms. Each of the five apps had a specific set of negating terms. Through an examination of the training data, a list of negating words and phrases were determined. Any instance that contained any of these words and phrases were excluded.
6. Exclude any instances wherein terms referring to experiencers who were not the subject (such as terms referring to family members or friends), appeared in the sentence. This was done through the determining a list of terms that could refer to an individual other than the patient (including terms for family members or friends or romantic partners). Any instance that contained one of these instances was excluded.
7. Exclude any instances where there were references to uncertainty about the diagnosis (as the aim of the application was to identify definite instances). This was done through creating a list of hedge words and excluding any instance that contained any of the hedge words.
8. Exclude instances of self-diagnosis (where the text indicates the patient diagnosed themselves with OCD). This was done through examining the training data, finding terms that were used in cases where self-diagnosis occurred and excluding those instances.

**Table 2:** List of terms used to identify candidates for exclusion.

Form	Negation	Other Experiencer	Self-Description	Hedge
c - obsessive compulsive	None	Mother/Father	Self-Described	Seem(s)
hoarded materials blocking passages	deny* (includes variations such as denied and denying)	Sister/Brother	He/She describe(s/d)	Possible* (Including variations such as possibility/and possibly
obsessions and compulsions. none	Nil	Parent	Described Him/Herself	Apparent(ly)
Obsessive Compulsive Index (including variations such as o.c.i, oci)	no(t) obses* (includes variations such as obsessed, obsessions and obsessional)	Son/Daughter	Say(s) that	Sound(s) like
	than (an) obses* (includes variations such as obsessed, obsessions and obsessional)	Sibling	told me	
	No History	Family		
	No Evidence	Boy/Girlfriend		
		Partner		
		Husband/Wife		
		qqqqq (a pseudonym for a family member or carer)		

We included lexical variances in the extraction rules [i.e. acronyms (e.g. OCD), misspellings (e.g. obses*instead of instead of obsessive)]. We took into account semantic variants in the terms of obsessive and compulsive in the extraction because, in so far as, these may have alternative meanings beyond their definitions in the context of OCD/OCS. This was done by distinguishing between the different examples of the obsessions and compulsions provided in the text. Through application of these rules, records were classified.

### Validation of an NLP App for extracting OCS

The validation dataset was used as a final test of how well the algorithm performed compared to manually coded data providing an indication of how well this algorithm would perform across the remaining 300,000 plus patient records on the CRIS system. To ensure that there was no information bias in the development of the application, the validation data remained unseen by the NLP developer (DC) throughout the App development process, until it was utilized to test the final version of the OCS algorithm. The accuracy of the OCS algorithm was evaluated using measurements of precision (i.e. positive predictive value) and recall (i.e. sensitivity) at the instance level. Precision was measured as the proportion of positive OCS instances identified by the NLP application tool that were correct according to the manual annotations of these same documents; recall was measured as the proportion of OCS instances in the documents (based on manual annotations) that were correctly identified by the NLP application tool. The development of the NLP application aimed to maximize precision in order to reduce the likelihood of false positive results. This NLP-based OCS application was then applied across the entire SLaM Case Register. Finally, for research purposes the OCS algorithm was combined with data from a pre-existing diagnosis algorithm and information on diagnosis from structured fields. Overall precision and recall produced by combining these approaches were calculated. The 95% confidence intervals were calculated using the exact binomial method^16^, which were calculated using the Stata software package.

## Results

Initially, a machine learning approach was trialed to develop an OCS NLP application because this could be developed and deployed more rapidly. This application was developed using TextHunter which is a bespoke piece of software developed at the SLaM BRC that allows for the fast creation and deployment of Machine Learning applications based on an annotated training set and gold standard. TextHunter utilizes an Support Vector Machine (SVM) approach to building an ML model. SVM development is seamlessly integrated into GATE, allowing for its smooth and rapid implementation. This approach had allowed for the creation of a wide range of successful applications using TextHunter. TextHunter develops models using a wide range for features and parameters, determining which one returns the highest Precision, Recall and F1 scores. However, the performance this approach was judged to be insufficient (attaining a precision of 0.74 and a recall of 0.51). It is possible that ML could return better results if a Deep Learning approach and been taken. However, while this was considered, its operation was determined to be too computationally and time intensive to be practical for the project. It does however remain an option for future exploration of the topic.

Examination of the text strings in the training dataset indicated that there was diversity in the words and phrases surrounding the key words in the training data set. Comparing the annotations generated by the algorithm to manual coding of the same training data indicated that the algorithm was not able to perform at a satisfactory level and that another approach was needed. It was noted that each of the individual keywords could form a potential application of its own. Therefore, we decided to build five separate component algorithms and combine them into a single functional OCS algorithm. This involved running each of the subset apps over the training data separately. The broad annotation rules that had been developed earlier were modified to suit each of the five component algorithms, individually. A decision was made, not to artificially use an equal number of keywords for each sub app, to ensure that the sample would be representative of overall data set with respect to the prevalence of those keywords.

The performance (precision and recall) of individual components of the OCS algorithm in the validation set and the performance overall across all text strings are described in Table 3. From the algorithm components themselves, performances of over 0.7 were observed in terms of precision except for Compulsions, Similarly, recall of over 0.8 was observed in each algorithm except for Ritual and Obsessions. In the specific context of OCD, the OCD component of the algorithm returned a precision of 1 and a recall of 0.85. The component algorithms were used together to detect the presence any OCS, (including OCD)–i.e. any text strings which described one or more of the following: obsessions, compulsion, OCD, hoarding, rituals–in accordance with the coding rules outlined in Table 4. The precision and recall (with 95% confidence intervals) for detecting any OCS, (including OCD) were 0.77 (0.65–0.86) and 0.67 (0.55–0.77) respectively.

**Table 3:** Performance of individual components of the OCS algorithm in the validation set (300 documents) and the performance overall for detecting any OCS (including OCD) across all strings with Precision (positive predictive value) and recall (sensitivity) provided.

Symptom	Precision	Recall
Obsessions	0.73	0.5
Compulsions	0.63	0.83
OCD	1	0.85
Hoard	0.73	0.81
Ritual	1	0.33
Any OCS (including OCD)	0.77	0.67

**Table 4:** Manual annotation rules for OCS.

**Negative for OCS**	Text makes no mention of OCS
	Text states that patient does not have OCS
	Text states that patient has either compulsions or obsessions, not both, and there is no information
	about any of the following:
	• Patient Distress
	• Obsessive or Compulsive symptoms described as egodystonic
	• Inability to stop Obsesions or Compulsions
	• Description of specific compulsions or specific obsessions
	• Patient Insight
	Text states that non-clinician observers (patient or family/friends) believe patient has obsessions
	or compulsions without describing YBOCS symptoms
	Text includes hedge words -i.e., possibly, apparently, seems -that specifically refer to OCS keywords
	Text includes risky, risk-taking, or self-harm behaviours
	Text includes romantic or weight-related (food related) words that modify OCS Keywords
**Positive for OCS**	Text states that patient has OCD features/OCD Symptoms
	Text states that patient has OCS
	Text includes hoarding, which was considered part of OCS, regardless of presence or absence of specific
	examples
	Text states that patient has at least 2 of the OCS keywords
	Text states that patient has either obsessive or compulsive or rituals or YBOCS and one of the following:
	• Obsessions or Compulsions are described as egodystonic
	• Intrusive, cause patient distress or excessive worrying/anxiety
	• Patient feels unable to stop obsessions or compulsions
	• Patient recognizes symptoms are irrational or senseless
	• Clinician provides specific YBOCS symptoms
	Text reports that patient has been diagnosed with OCD by clinician

## Discussion

These results highlight that it is feasible to develop a tool to address a relatively complex data classification task in electronic health records using NLP. The algorithm we developed was able to identify co-morbid OCS in people diagnosed with schizophrenia, schizoaffective disorder or bipolar disorder with acceptable precision and recall, particularly in terms of the ability to identify OCD, as compared to existing algorithms that have been developed for CRIS (some of which are illustrated in a previous publication^7^) which showed a range of precisions between 0.93 to 0.97 and a range of recalls between 0.59 to 0.99.

Identifying OCS using clinical records is arguably a comparatively challenging task for an NLP application for a number of reasons. Unlike many other health constructs (e.g. hypertension) OCS are often described using terms which have a wide range of usage in both specialized and lay contexts. For example, ‘obsession’ may be used to describe nothing more than a keen interest, or ‘compulsion’ to describe the desire to engage in risk taking behaviours, such as gambling, neither of which would qualify as OCS. Also, these symptoms can manifest in a variety of ways and in many cases, may not be the primary concern of the clinicians writing clinical notes and correspondence. Moreover, OCS need to be clinically distinguishable from schizophrenic mannerisms or posturing or other psychosis related repetitive thoughts or behaviour^10^ consequently the algorithm needed to be able to use information presented in the free text to make these distinctions.

To the best of our knowledge, this is the first time an algorithm has been developed to extract OCS from free text using NLP. However, NLP or other algorithms have been used to extract other types of information from clinical records. Examples of this are applications to find the presence of cognitive behavioural therapy (CBT) delivery^17^, adverse drug effects (ADR)^18^ and antipsychotic polypharmacy data^13^.

A key strength of the OCS algorithm was its ability to successfully identify obsessive and compulsive symptoms in free text, with a reasonable level of precision despite the complexity of these texts and the variety of way clinicians refer to these symptoms. The performance of this algorithm improved when divided into component algorithms and modified accordingly, performing particularly well at identifying OCD. The key limitation was the lower recall, which created a risk of underestimating cases of OCS. In the development of this algorithm, precision was considered more important than recall, i.e. false positives were considered to be a more important issue to avoid than false negatives. The results presented here are for instances in the text describing OCS (including OCD). In practice this NLP algorithm would be applied to classify patients where there are likely to be multiple instances describing the same symptoms. Consequently, OCS instances that are missed (due to the lower recall) may be less important because there are likely to be other instances for the same patient which the algorithm will detect.

To develop this algorithm, we undertook a rules-based rather after initially trying a machine learning approach. A rules based approach has its own limitations. Firstly, it is a substantially more time-consuming approach than a machine learning one^19^. This is because it takes a coder far longer to identify and code rules than for an automated system to construct a model. Furthermore, a rules-based approach is far more vulnerable to a coder’s subjectivity and personal biases than a machine learning one. This is a particularly key issue given the importance in NLP in understanding the intent of the text that is analyzed. By comparison, the downside to a machine learning approach is that its efficacy decreases in relation to the complexity of the task which may mean the algorithm is unable to perform well or that the algorithm would require an increasingly large set of training data (increasing the time taken to process that data and create the application and the time required to develop the training data)^4^.

There are a number of avenues for further development. One approach is to endeavour to improve algorithm performance by increasing the size of the training dataset providing further examples of positive and negative instances which would allow new rules to be developed. A further point of development is identifying the temporality around positive mentioned OCS, which is not currently determined. This is an important challenge, as there have not been any previously developed methods for determining (from unstructured text), if the subject of a record currently has OCS or if they had them in the past (and if so how far in the past). Therefore, future work will involve adding a temporality component. In addition, the work that was done was limited through using a single corpus (CRIS), future work will involve using the algorithm over the text data of clinical records from other trusts, which will give an indication of the algorithms generalizability.
